# Preserved transcriptional networks in immune signaling pathways associated with chronic disease identified in Alzheimer’s disease and Parkinson’s disease, cross‐tissue analysis

**DOI:** 10.1002/alz.092349

**Published:** 2025-01-03

**Authors:** Samantha L Strickland, Wei Tsai, Xuan Chen, Yesesri Cherukuri, Mariet Allen, Zachary Quicksall, Xue Wang, Kejal Kantarci, Minerva M. Carrasquillo, Kwangsik Nho, Andrew J. Saykin, Ronald C. Petersen, Joseph S. Reddy, Nilüfer Ertekin‐Taner

**Affiliations:** ^1^ Mayo Clinic, Jacksonville, FL USA; ^2^ Department of Radiology, Mayo Clinic, Rochester, MN USA; ^3^ Center for Computational Biology and Bioinformatics, Indiana University School of Medicine, Indianapolis, IN USA; ^4^ Department of Radiology and Imaging Sciences, Indiana University School of Medicine, Indianapolis, IN USA; ^5^ Department of Medical and Molecular Genetics, Indiana University School of Medicine, Indianapolis, IN USA; ^6^ Center for Neuroimaging, Department of Radiology and Imaging Sciences, Indiana University School of Medicine, Indianapolis, IN USA; ^7^ Department of Neurology, Mayo Clinic, Rochester, MN USA

## Abstract

**Background:**

Systemic inflammation plays a pivotal role in many chronic diseases including Alzheimer’s disease (AD). Assessing the composition of immune pathways in neurodegenerative diseases can contribute to precision medicine. Using publicly available transcriptomic data, we sought to elucidate transcriptional networks pertinent to inflammatory pathways across brain regions and peripheral blood in AD/mild cognitive impairment (MCI) and peripheral blood in Parkinson’s disease (PD).

**Method:**

For the AD/MCI vs. control dataset, we analyzed bulk‐RNAseq collected from 6 brain regions of donors from ROSMAP, Mayo Clinic, and Mount Sinai School of Medicine (MSSM) brain banks available from the AMP‐AD consortium. Ante‐mortem, blood RNAseq expression data was retrieved from the AMP‐AD Emory Vascular cohort and Mayo Clinic Study of Aging (MCSA). We also collected blood‐derived microarray expression data from the Alzheimer’s Disease Neuroimaging Initiative (ADNI). For the PD vs Control dataset, blood‐derived bulk‐RNAseq from the PDBP and PPMI cohorts were available through the AMP‐PD consortium. Following quality control, normalization, and residual generation to account for biological and technical variables, co‐expression network modules and their enriched pathways were identified using WGCNA within each dataset. Module/trait correlation tests for aging and diagnosis (cases [AD/MCI or PD] vs control) phenotypes were evaluated. Gene Ontology enrichment analyses were executed to identify enriched pathways and brain or blood cell types within the modules. Modules were tested for preservation across cohorts.

**Result:**

We identified conserved immune signatures across brain regions and cohorts. Modules involved in immune response were preserved across all cohorts. Blood consensus modules involved in immune response were preserved in the brain and vice versa. Some immune modules were associated with AD/MCI, PD, and/or aging. Brain immune modules are significantly associated with aging and/or AD. Significant correlations (q<0.05) with PD diagnosis were present. In the MCSA and Emory vascular cohorts, there were no significant (q<0.05) associations between modules and diagnosis, while in ADNI there were nominal (p<0.05) associations.

**Conclusion:**

Preserved transcriptional immune networks were identified across blood and brain and across two neurodegenerative diseases. Expanding gene co‐expression network analyses to other diseases and integrating additional omics measures and phenotypes can further strengthen these findings to unravel the immune signatures across complex diseases.

